# The Prognostic Value of C-reactive Protein to Albumin Ratio in Patients with Isolated Degenerative Aortic Valve Stenosis Undergoing Surgical Aortic Valve Replacement

**DOI:** 10.21470/1678-9741-2019-0114

**Published:** 2020

**Authors:** Serkan Kahraman, Arda Can Dogan, Gokhan Demirci, Ali Riza Demir, Emre Yilmaz, Hicaz Zencirkiran Agus, Ali Kemal Kalkan, Fatih Uzun, Mehmet Erturk

**Affiliations:** 1 Department of Cardiology, University of Health Sciences, Istanbul Mehmet Akif Ersoy Thoracic and Cardiovascular Surgery Center, Training and Research Hospital, Istanbul, Turkey.

**Keywords:** Albumin, Aortic Valve Stenosis, C-Reactive Protein, Heart Valve Prosthesis, Hospitalization, Reoperation

## Abstract

**Objective:**

To evaluate the prognostic value of C-reactive protein to albumin ratio (CAR) in patients with severe aortic valve stenosis undergoing surgical aortic valve replacement (AVR).

**Methods:**

Four hundred seventy-six patients with severe degenerative aortic stenosis who underwent successful isolated surgical AVR were enrolled. Hospitalization due to heart failure, surgical aortic reoperation, paravalvular leakage rates, and long-term mortality were evaluated in the whole study group. The participants were divided into two groups, as 443 patients without mortality (group 1) and 33 patients with mortality (group 2) during the follow-up time.

**Results:**

CAR was lower in patients without mortality than in those with mortality during the follow-up time (0.84 [0.03-23.43] *vs*. 2.50 [0.22-26.55], respectively, *P*<0.001). Age (odds ratio [OR]: 1.062, confidence interval [CI]: 1.012-1.114, *P*=0.014), CAR (OR: 1.221, CI: 1.125-1.325, *P*<0.001), ejection fraction (OR: 0.956, CI: 0.916-0.998, *P*=0.042), and valve type (OR: 2.634, CI: 1.045-6.638, *P*=0.040) were also found to be independent predictors of long-term mortality. Additionally, rehospitalization (0.86 [0.03-26.55] *vs*. 1.6 [0.17-24.05], *P*=0.006), aortic reoperation (0.87 [0.03-26.55] *vs*. 1.6 [0.20-23.43], *P*=0.016), and moderate to severe aortic paravalvular leakage (0.86 [0.03-26.55] *vs*. 1.86 [0.21-19.50], *P*=0.023) ratios were associated with higher CAR.

**Conclusion:**

It was firstly described that CAR was strongly related with increased mortality rates in patients with isolated severe aortic stenosis after surgical AVR. Additionally, rehospitalization, risk of paravalvular leakage, and aortic reoperation rates were higher in patients with increased CAR than in those without it.

**Table:** 

Abbreviations, acronyms & symbols				
ACEI	= Angiotensin-converting enzyme inhibitor		IL-6	= Interleukin-6
ARB	= Angiotensin receptor blocker		LDL	= Low-density lipoprotein
AVR	= Aortic valve replacement		LVEDD	= Left ventricular end-diastolic diameter
CAR	= C-reactive protein to albumin ratio		LVESD	= Left ventricular end-systolic diameter
CI	= Confidence interval		NYHA	= New York Heart Association
COPD	= Chronic obstructive pulmonary disease		OR	= Odds ratio
CRP	= C-reactive protein		SPSS	= Statistical Package for Social Sciences
EF	= Ejection fraction		TG	= Triglyceride
HDL	= High-density lipoprotein			

## INTRODUCTION

Aortic stenosis is the most common form of degenerative valvular heart disease, with increasing prevalence, and it is still the leading cause of surgical valve replacement therapy, especially in developing countries^[1]^. Although several etiological risk factors of aortic stenosis have been described, it is known that degenerative valvular stenosis is an active process with inflammatory histological valve changes, like atherosclerotic plaques^[2]^. Thus, the chronic inflammatory process plays an important role on atherosclerosis, an uncontested underlying pathogenic mechanism of degenerative valvular aortic stenosis.

High-sensitive C-reactive protein (CRP) is one of the most specific biomarkers of systemic inflammation. It was demonstrated that serum CRP levels predict progression and severity of aortic stenosis due to the pathogenic role of inflammation on valvular disease^[3]^. Contrary to CRP, albumin is reduced in the chronic inflammatory process, as a negative acute phase responder^[4]^. Accordingly, CRP to albumin ratio (CAR) is a more sensitive marker to predict inflammation than CRP or albumin rates alone, due to its two different directions (increased CRP and decreased albumin). While inflammatory markers have been evaluated on severity and prognosis of aortic stenosis, they have not been adequately studied to determine their effect on the prognosis after aortic valve replacement (AVR) therapy. We aimed to evaluate the prognostic value of CAR on clinical events in patients with isolated severe degenerative aortic stenosis undergoing surgical AVR.

## METHODS

### Study Population

This retrospective observational study was conducted at a tertiary high-volume center in Turkey from January 2010 to December 2017. Eight hundred seventy-six patients with severe degenerative aortic stenosis who underwent successful surgical AVR were observed. After exclusion, four hundred seventy-six (476) patients were enrolled in this study. Patients undergoing urgent surgery due to acute aortic regurgitation and patients with active infection, with known coronary artery disease, undergoing percutaneous coronary intervention, with previously or simultaneously coronary artery bypass grafting or cardiac valve replacement surgery other than aortic valve, with type A aortic dissection, malignancy, or end-stage hepatic or renal disease were excluded from the study. Patients with aortic mismatch after operation were also excluded from the study. The participants were divided into two groups, as patients without mortality (group 1) and with mortality (group 2) during the follow-up time. The study was approved by the local ethical committee.

### Biochemical Analysis

All biochemical analyses were measured before performing AVR. Total cholesterol, low-density lipoprotein (LDL) cholesterol, high-density lipoprotein (HDL) cholesterol, triglyceride (TG), fasting blood glucose, blood urea nitrogen, creatinine, and complete blood count were measured after stopping the oral intake for eight to 12 hours and blood samples were drawn from the brachial veins. CRP and serum albumin levels were measured by using The Roche Diagnostics Cobas® 8000 c502 analyzer (Indianapolis, United States of America). The value of 0.0-5.0 mg/l was accepted as normal range for CRP and the value of 3.5-5.2 g/dl was accepted for serum albumin.

### Clinical Follow-up

To evaluate the effect of low and high levels of CAR in patients with severe aortic stenosis undergoing surgical AVR on long-term clinical events, including hospitalization due to heart failure, surgical aortic reoperation and all-cause death and mortality were compared. Patients’ follow-up visits were done at hospital admission for suitable patients, and telephone follow-up visits were done for others.

### Statistical Analysis

Statistical analysis was made using the Statistical Package for Social Sciences (IBM SPSS Statistics for Windows, version 21.0, released in 2012, IBM Corp., Armonk, New York, United States of America) computer software. Data were expressed as “n (%)” for categorical variables; Pearson’s chi-square and Fisher’s exact tests were performed for categorical variables. After fitness to normal distribution was analyzed with the Kolmogorov-Smirnov test, data were expressed as “median (minimum-maximum)” for abnormal distribution. Mann-Whitney U test was used for comparing quantitative variables with abnormal distribution. Univariate and multivariate logistic regression analyses were used to determine the independent predictors of mortality after surgical AVR. A *P*-value < 0.05 was considered statistically significant.

## RESULTS

Basal demographic and clinical variables of the whole study group were demonstrated in Table 1. Patients without mortality during the follow-up time formed group 1 and patients with mortality formed group 2. There were no significant differences in gender; smoking status; diabetes mellitus; hypertension; dyslipidemia; peripheral arterial disease; atrial fibrillation presence; chronic obstructive pulmonary disease; previous cerebrovascular disease; history of congestive heart failure; symptoms such as dyspnea, angina, and syncope; levels of leukocyte, thrombocyte, total cholesterol, LDL cholesterol, HDL cholesterol, TG, and albumin; medication usage (beta-blocker, calcium channel blocker, and statin); aortic valve area; maximum and mean aortic gradients; left ventricular end-diastolic and -systolic diameters; and postoperative hospital discharging time between the groups.

**Table 1:** Basal demographic and clinical variables of both groups with mortality absence and presence during the follow-up time.

		Mortality absence Group 1 (n=443)	Mortality presence Group 2 (n=33)	*P*-value
Age (years)		61 (40-88)	74 (46-86)	<0.001
Gender (female) % (n)		31.4 (139)	45.5 (15)	0.095
Smoking % (n)		25.1 (111)	21.2 (7)	0.622
Diabetes mellitus % (n)		23.9 (106)	24.2 (8)	0.967
Hypertension % (n)		49.7 (220)	39.4 (13)	0.255
Dyslipidemia % (n)		28.4 (126)	27.3 (9)	0.886
Peripheral arterial disease % (n)		2.9 (13)	6.1 (2)	0.279
Atrial fibrillation % (n)		8.4 (37)	12.1 (4)	0.314
COPD % (n)		14.7 (65)	9.1 (3)	0.277
Previous cerebrovascular disease % (n)		0.2 (1)	3 (1)	0.134
Previous congestive heart failure % (n)		14.9 (66)	27.3 (9)	0.060
Dyspnea % (n)	NYHA I-II	44.2 (196)	27.3 (9)	0.058
	NYHA III-IV	55.8 (247)	72.7 (24)	
Angina % (n)		50.3 (223)	60.6 (20)	0.255
Syncope % (n)		9.9 (44)	15.2 (5)	0.244
Creatinine (mg/dl)		0.86 (0.40-8.60)	1.0 (0.50-4.60)	0.003
Hemoglobin (g/dl)		13.2 (6.6-18.0)	11.9 (8.1-16.5)	0.011
Leukocytes × 10^3^/mm^3^		7.9 (2.1-24.5)	8.1 (4.0-27.7)	0.699
Thrombocyte × 10^3^/mm^3^		234 (37-565)	231 (101-380)	0.376
Total cholesterol (mg/dl)		178 (67-330)	172 (118-282)	0.859
LDL cholesterol (mg/dl)		106 (10-225)	101 (63-188)	0.824
HDL cholesterol (mg/dl)		42 (9-111)	48 (17-110)	0.324
Triglyceride (mg/dl)		121 (16-613)	140 (53-800)	0.496
CRP (mg/l)		3.5 (0.3-82.0)	10.0 (1.0-93.8)	<0.001
Albumin (g/dl)		4.2 (2.1-37.6)	4.2 (1.7-4.9)	0.445
CRP to albumin ratio		0.84 (0.03-23.43)	2.50 (0.22-26.55)	<0.001
Medication usage % (n)	Beta-blocker	80.8 (358)	66.7 (22)	0.051
	ACEI or ARB	43.8 (194)	18.2 (6)	0.004
	Calcium channel blocker	13.3 (59)	12.1 (4)	0.551
	Statin	22.1 (98)	18.2 (6)	0.597
Ejection fraction (%)		60 (15-70)	55 (25-65)	0.001
Aortic valve area (cm^2^)		0.7 (0.3-0.9)	0.7 (0.4-0.9)	0.234
Maximum gradient (mmHg)		76 (40-181)	74 (43-154)	0.667
Mean gradient (mmHg)		50 (25-119)	46 (26-90)	0.220
LVEDD (mm)		50 (33-85)	50 (38-68)	0.525
LVESD (mm)		32 (19-78)	33 (20-59)	0.953
Left atrial diameter (mm)		39 (22-59)	42 (28-52)	0.012
Valve type % (n)	Bioprosthetic valve	29.1 (129)	57.6 (19)	0.001
	Mechanical prosthetic valve	70.9 (314)	42.4 (14)	
Postoperative discharging time (days)		9 (5-80)	9 (5-64)	0.517
Follow-up time (months)		51 (2-300)	72 (1-267)	<0.001

ACEI=angiotensin-converting enzyme inhibitor; ARB=angiotensin receptor blocker; COPD=chronic obstructive pulmonary disease; CRP=C-reactive protein; HDL=high-density lipoprotein; LDL=low-density lipoprotein; LVEDD=left ventricular end-diastolic diameter; LVESD=left ventricular end-systolic diameter; NYHA=New York Heart Association

**Fig. 1:**
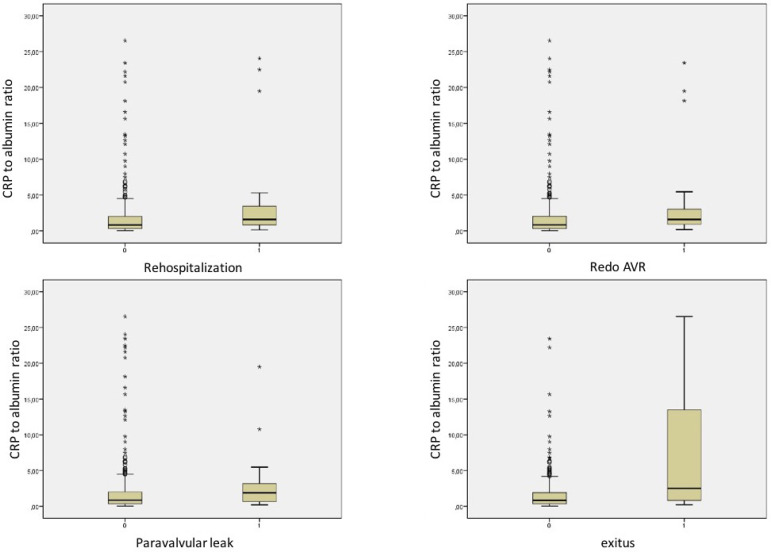
Comparison of serum CRP to albumin ratio in the whole study group according to rehospitalization, aortic reoperation, paravalvular leakage, and mortality rates. AVR=aortic valve replacement; CRP=C-reactive protein

Patients without mortality (group 1) were younger than patients with mortality (group 2) (61 [40-88] years *vs*. 74 [46-86] years, respectively, *P*<0.001). While creatinine (0.86 [0.40-8.60] *vs*. 1.0 [0.50-4.60], *P*=0.003) and CRP (3.5 [0.3-82.0] *vs*. 10.0 [1.0-93.8], *P*<0.001) levels in group 1 were lower than in group 2, hemoglobin level was higher in group 1 than in group 2 (13.2 [6.6-18.0] *vs*. 11.9 [8.1-16.5], *P*=0.011). Angiotensin-converting enzyme inhibitor (ACEI) or angiotensin receptor blocker (ARB) usage rate was higher in group 1 than in group 2 (43.8% [194] *vs*. 18.2% [6], *P*=0.004). Ejection fraction (EF) was also higher in group 1 than in group 2 (60 [15-70] *vs*. 55 [25-65], *P*=0.001). However, left atrial diameter was narrower in group 1 than in group 2 (39 [22-59] *vs*. 42 [28-52], *P*=0.012). Mechanical prosthetic valve ratio was higher in group 1 than in group 2 (70.9% [314] *vs*. 42.4% [14], *P*=0.001). And CAR was lower in group 1 than in group 2 (0.84 [0.03-23.43] *vs*. 2.50 [0.22-26.55], *P*<0.001) (Figure 1). Additionally, follow-up time after surgical AVR was shorter in group 1 than in group 2 (51 [2-300] months vs. 72 [1-267] months, *P*<0.001).

In multivariate logistic regression analysis, age (odds ratio [OR]: 1.062, confidence interval [CI]: 1.012-1.114, P=0.014), CAR (OR: 1.221, CI: 1.125-1.325, *P*<0.001), EF (OR: 0.956, CI: 0.916-0.998, *P*=0.042), and valve type (OR: 2.634, CI: 1.045-6.638, *P*=0.040) were found to be independent predictors of long-term mortality in patients with severe aortic stenosis undergoing surgical AVR (Table 2).

**Table 2:** Univariate and multivariate logistic regression analyses giving information about independent predictors of mortality.

	Univariate analysis			Multivariate analysis		
	Odds ratio	95% CI (lower-upper)	*P*-value	Odds ratio	95% CI (lower-upper)	*P*-value
Age	1.075	1.038-1.113	<0.001	1.062	1.012-1.114	0.014
Gender (female)	0.549	0.269-1.120	0.099			
Smoking	1.242	0.525-2.940	0.622			
Diabetes mellitus	0.983	0.431-2.244	0.967			
Hypertension	1.518	0.737-3.126	0.258			
Dyslipidemia	1.060	0.479-2.343	0.886			
Peripheral arterial disease	0.469	0.101-2.170	0.332			
Atrial fibrillation	0.661	0.220-1.981	0.460			
COPD	1.720	0.510-5.799	0.382			
Previous cerebrovascular disease	0.072	0.004-1.185	0.066			
Previous congestive heart failure	0.467	0.208-1.049	0.065			
Dyspnea	2.116	0.962-4.656	0.063			
Angina	0.659	0.320-1.357	0.258			
Syncope	0.618	0.227-1.681	0.345			
Creatinine	1.544	1.044-2.283	0.029	1.212	0.747-1.966	0.436
Hemoglobin	0.811	0.690-0.953	0.011	1.089	0.883-1.342	0.427
Leukocytes	1.032	0.912-1.167	0.621			
Thrombocyte	0.997	0.992-1.002	0.268			
Total cholesterol	1.001	0.993-1.008	0.845			
LDL cholesterol	0.999	0.989-1.008	0.779			
HDL cholesterol	1.015	0.990-1.040	0.236			
Triglyceride	1.003	0.999-1.006	0.151			
CRP to albumin ratio	1.242	1.157-1.334	<0.001	1.221	1.125-1.325	<0.001
Ejection fraction	0.955	0.926-0.984	0.003	0.956	0.916-0.998	0.042
LVEDD	0.984	0.941-1.030	0.490			
LVESD	1.008	0.971-1.047	0.675			
Left atrial diameter	1.066	1.007-1.129	0.029	0.977	0.903-1.057	0.558
Postoperative discharging time	1.039	1.012-1.067	0.005	1.033	0.998-1.069	0.067
Valve type	3.303	1.608-6.788	0.001	2.634	1.045-6.638	0.040

CI=confidence interval; COPD=chronic obstructive pulmonary disease; CRP=C-reactive protein; HDL=high-density lipoprotein; LDL=low-density lipoprotein; LVEDD=left ventricular end-diastolic diameter; LVESD=left ventricular end-systolic diameter

Additionally, rehospitalization (0.86 [0.03-26.55] *vs*. 1.6 [0.17-24.05], *P*=0.006), aortic reoperation (0.87 [0.03-26.55] *vs*. 1.6 [0.20-23.43], *P*=0.016), and moderate to severe aortic paravalvular leakage (0.86 [0.03-26.55] *vs*. 1.86 [0.21-19.50], *P*=0.023) ratios were associated with higher CAR (Table 3 and Figure 1).

**Table 3:** Comparison of CRP to albumin ratio in the presence and absence of rehospitalization, aortic reoperation, and moderate to severe paravalvular leak.

	**Rehospitalization absence**	**Rehospitalization presence**	***P*-value**
CRP to albumin ratio	0.86 (0.03-26.55)	1.6 (0.17-24.05)	0.006
	**Aortic reoperation absence**	**Aortic reoperation presence**	*P*-value
CRP to albumin ratio	0.87 (0.03-26.55)	1.6 (0.20-23.43)	0.016
	**None to mild paravalvular leak**	**Moderate to severe paravalvular leak**	***P*-value**
CRP to albumin ratio	0.86 (0.03-26.55)	1.86 (0.21-19.50)	0.023

CRP=C-reactive protein

## DISCUSSION

In our retrospective study, to the best of our knowledge, it was firstly demonstrated that higher CAR was found to be strongly associated with increased mortality rates in patients with severe degenerative aortic stenosis undergoing surgical AVR. Additionally, hospitalization rates due to heart failure after AVR were related with higher CAR. Surgical aortic reoperation and moderate to high paravalvular leakage ratios were also associated with higher CAR.

Inflammation is known as an important risk factor for cardiovascular diseases^[5]^. Histopathological findings of inflammation including calcification, fibrosis, and lipid storage have been demonstrated in degenerative valvular aortic stenosis, like atherosclerosis^[2]^. Due to the mentioned reason, several biochemical markers have been described to detect inflammation on progression and prognosis of aortic stenosis. CRP was firstly described by Tillet and Francis as a marker of ongoing inflammation. In the event of chronic inflammatory process, CRP, which is produced in the liver by interleukin-6 (IL-6), tumor necrosis factor α, and other pro-inflammatory cytokines, has also been localized in the aortic valve tissue, as well as T lymphocytes and macrophage infiltration and oxidized lipoproteins^[6]^. Increased CRP causes expression of adhesion molecules and plasminogen activator inhibitor-1 and diminishes nitric oxide production, resulting in vasoconstriction, pro-thrombotic, and pro-inflammatory statuses linked to endothelial dysfunction^[7,8]^. In the course of chronic inflammation, valvular degeneration and stenosis can be seen and its progression is also related with the inflammatory status. Valvular stenosis occurs more rapidly in case of increased inflammation. Thus, higher level of CRP causes valvular aortic degeneration and makes it stenosed. Additionally, serum CRP level is found to be correlated with valvular expression. It means that increased serum CRP levels are related with progression, severity, and prognosis of aortic stenosis. Skowasch et al.^[9]^ demonstrated that CRP localized in the aortic valve tissue of patients with aortic stenosis is correlated with serum CRP levels and it was revealed that serum CRP level is associated with progression of aortic stenosis and long-term survival in patients with asymptomatic aortic stenosis^[3]^. As for albumin, it is also produced in the liver and it has an antioxidant activity^[10]^. Contrary to CRP, albumin is reduced in the chronic inflammatory process, as a negative acute phase response protein by inflammatory substances, including IL-6^[4]^. The decrease in the serum albumin level results in the increase blood viscosity and disruption in endothelial functions^[11]^.

Acute phase reactants’ responses do not have to be in a similar degree for each inflammatory status. Different inflammation-based prognostic scores were identified to provide more stable regulation due to this reason. CAR, as a single inflammatory index, provides the stability against the CRP and albumin alone. In previous studies, CAR had better valuable prognostic factor to reflect inflammation status in different inflammatory diseases. Fairclough et al.^[12]^ demonstrated that CAR had a favorable prognostic value in elderly patients in acute exacerbations of chronic disease and that CAR was a better marker than CRP alone to predict long-term mortality in patients at intensive care units^[13]^. Additionally, similar results have been demonstrated in patients with malignancies, vasculitis, and critically ill patients^[14-16]^. CAR was also studied in cardiovascular diseases, especially in atherosclerotic coronary artery disease. Coronary artery disease severity was found to be associated with higher CAR values in patients with stable coronary artery disease and acute coronary syndrome^[17,18]^. This could be explained by the higher inflammatory status linked to extend of the coronary atherosclerosis. Furthermore, it was demonstrated that increased CAR predicted poor prognosis in patients with ST-segment elevation myocardial infarction^[19]^.

In the light of foregoing data, increased inflammation makes the cardiac endothelium critically ill and it affects the valvular tissue, *e.g*., coronary atherosclerosis. Thus, cardiac valvular degeneration can be seen more distinctly, and its progression and prognosis can be worse. Unsurprisingly, in our study, it was demonstrated that higher inflammatory status was associated with poor prognosis. It is known that adverse clinical events are seen more explicitly in case of chronic inflammation^[12-16]^, so that higher inflammation as the underlying mechanism of the chronic degenerative valvular disease causes increased mortality rates even if the valvular disease is treated. In previous studies, increased CRP value was found to be related with poor prognosis in patients with severe aortic stenosis^[3]^. However, to the best of our knowledge, our study was the first to demonstrate that higher inflammatory status affected long-term mortality in patients with severe degenerative aortic stenosis undergoing surgical AVR. It could be explained by some possible underlying mechanisms. Firstly, left ventricular myocardium is affected by inflammation. Although surgical treatment of aortic stenosis is performed successfully, sufficient and completely remodeling on left ventricle cannot be seen. Thus, large inflammatory burden causes impaired left ventricular function, resulting in heart failure. It was supported in previous studies in which inflammation was found to be related with heart failure^[20]^. In conclusion, increased hospitalization and mortality rates can be seen due to irreversible heart failure. Supporting this, in our study, hospitalization rates after surgical AVR were found to be increased in patients with higher CAR. Secondly, inflammatory status also predicts surgical success rates. In these patients, surgical suture and prosthetic valve can become degenerative due to increased inflammatory burden. Thus, postoperative complications such as paravalvular leakage and pannus formation can affect adverse cardiac events in patients with higher inflammatory status. We also found out that paravalvular leakage ratio was higher in patients with increased CAR. While it can also be related with surgical suture technique, in this single-center population, this reason cannot affect our outcomes. Consequently, the mentioned higher CAR linked to failure of surgical treatment can result in increased aortic reoperation rates. Additionally, inflammation linked to increased risk of arrhythmia could be the other possible underlying reason of adverse clinical outcomes in this study population. Unfortunately, in our study, clinical arrhythmic events were not evaluated.

Left ventricular EF is of importance to predict adverse cardiac events and mortality in patients with aortic stenosis. It is also a determinant factor for treatment time and modality. After surgical replacement therapy, higher mortality rates may be seen in patients with left ventricular systolic dysfunction. Taniguchi et al.^[21]^ revealed that impaired EF was related with increased mortality rates in 3815 patients with severe aortic stenosis. Supporting these, lower EF was an independent predictor of mortality in our study. Age is another risk factor for poor prognosis in patients with valvular heart disease. Hussain et al.^[22]^ demonstrated that age was a predictor of mortality in patients with severe asymptomatic aortic stenosis undergoing surgical AVR. Also, it is known that mechanical prosthetic valves are more durable than bioprosthetic valves. Thus, bioprosthetic valves are commonly preferred in the ageing population. In our study, older age and bioprosthetic valves were found to be independent predictors of mortality due to the mentioned reasons. Additionally, ACEI or ARB usage causes decreased adverse cardiac outcomes, such as mortality, in patients with cardiovascular disease. In a meta-analysis, it was shown that treatment with ACEI or ARB was better than placebo in reducing all-cause mortality, especially in patients with chronic heart failure with reduced EF^[23]^. It was supported in our study by lower ACEI or ARB usage ratio in patients with mortality after surgical AVR therapy.

In the light of the mentioned data, CAR is an easy, cheap, and rapid prognostic risk score to detect high-risk patients for surgical AVR. However, large scaled studies are needed to evaluate the impact of CAR on adverse clinical events in patients with severe aortic stenosis after surgical AVR.

### Limitations

The major limitation of this study was a relatively small sample size to evaluate the long-term mortality. Lack of other major adverse cardiac events, such as progression of arrhythmia and cerebrovascular disease, was the other limitation. Unknown etiology of mortality in the study group due to retrospectively follow-up visits was also another crucial limitation.

## CONCLUSION

Inflammatory process is known as a risk factor of cardiovascular diseases. However, in our study, it was firstly described that higher CAR was strongly related with increased mortality rates in patients with isolated severe degenerative aortic stenosis after surgical AVR. In addition to this, rehospitalization rates due to heart failure, risk of paravalvular leakage, and aortic reoperation rates were higher in patients with increased CAR. In conclusion, CAR is an easy, cheap, and rapid prognostic risk score to detect high-risk patients for surgical AVR.

**Table:** 

Author's roles & responsibilities	
SK	Substantial contributions to the conception or design of the work; or the acquisition, analysis, or interpretation of data for the work; drafting the work or revising it critically for important intellectual content; final approval of the version to be published
ACD	Drafting the work or revising it critically for important intellectual content; agreement to be accountable for all aspects of the work in ensuring that questions related to the accuracy or integrity of any part of the work are appropriately investigated and resolved; final approval of the version to be published
GD	Drafting the work or revising it critically for important intellectual content; agreement to be accountable for all aspects of the work in ensuring that questions related to the accuracy or integrity of any part of the work are appropriately investigated and resolved; final approval of the version to be published
ARD	Substantial contributions to the conception or design of the work; or the acquisition, analysis, or interpretation of data for the work; drafting the work or revising it critically for important intellectual content; final approval of the version to be published
EY	Substantial contributions to the conception or design of the work; or the acquisition, analysis, or interpretation of data for the work; drafting the work or revising it critically for important intellectual content; final approval of the version to be published
HZA	Drafting the work or revising it critically for important intellectual content; agreement to be accountable for all aspects of the work in ensuring that questions related to the accuracy or integrity of any part of the work are appropriately investigated and resolved; final approval of the version to be published
AKK	Substantial contributions to the conception or design of the work; or the acquisition, analysis, or interpretation of data for the work; drafting the work or revising it critically for important intellectual content; final approval of the version to be published
FU	Substantial contributions to the conception or design of the work; or the acquisition, analysis, or interpretation of data for the work; drafting the work or revising it critically for important intellectual content; final approval of the version to be published
ME	Substantial contributions to the conception or design of the work; or the acquisition, analysis, or interpretation of data for the work; drafting the work or revising it critically for important intellectual content; final approval of the version to be published
